# Mental health in Italy after two years of COVID-19 from the perspective of 1281 Italian physicians: looking back to plan forward

**DOI:** 10.1186/s12991-022-00410-5

**Published:** 2022-08-10

**Authors:** Alessandro Cuomo, Mario Amore, Maria Felice Arezzo, Sergio De Filippis, Alessandra De Rose, Silvestro La Pia, Alessandro Pirani, Riccardo Torta, Andrea Fagiolini

**Affiliations:** 1 Department of Molecular Medicine, University of Siena, Siena, Italy; 2 Section of Psychiatry, Department of Neuroscience, Ophthalmology, Genetics and Infant-Maternal Science, Istituti di Ricovero e Cura a Carattere Scientifico (IRCCS) Ospedale Policlinico San Martino, University of Genoa, Genoa, Italy; 3 Department of Methods and Models for Economy, Territory, and Finance – Sapienza Università di Roma, Roma, Italy; 4 Neuropsychiatric Clinic, Villa Von Siebenthal, Genzano di Roma, Italy; 5 Dipartimento di Salute Mentale, ASL Napoli 3 Sud, Napoli, Italy; 6 Center for Cognitive Disorders and Dementia, Health County of Ferrara, Cento, Italy; 7 Department of Neuroscience, University of Turin, Turin, Italy

**Keywords:** COVID-19, Mental health, Anxiety, Agitation, Physician

## Abstract

**Background:**

The COVID-19 pandemic has generated an unprecedented global crisis that is profoundly affecting mental health and mental health care. The aim of this study was to survey a relatively large group of Italian physicians about their perceived impact of COVID-19 on the mental health of the Italian population and about their suggestions on the best strategies to address the current and future challenges.

**Methods:**

One thousand two hundred eighty-one (1,281) physicians were surveyed between November 2021 and February 2022.

**Results:**

Eighty-one percent of respondents reported an increase in the number of people with mental illness presenting to their practice during the COVID-19 pandemic. Thirty-four percent reported a 26–50% increase in the number of people with mental illness in their community; approximately 33% reported a 1–25% increase; and 26.9% reported a 51–75% increase. The most commonly reported mental issues that increased because of COVID-19 were agitation, mood and anxiety disorders. Regarding the suggested strategies to address future challenges related to the COVID-19 pandemic, 34.6% of respondents recommended providing psychoeducation to the general population for early detection of mental illness and developing strategies to reduce the impact of COVID-19-related stress. In addition, 12.6% of respondents suggested improving telehealth services, while 12.3% suggested the need for increased funding for community-based care. When asked about physicians' opinion on the possibility of an increased prevalence of mental illness in the next 12 months, more than 30% of them predicted an increase in stress-related illnesses, while 25.2% were more concerned about a worsening of the ongoing clinical conditions of patients with previous psychiatric disorders. However, 21% of respondents believed that people's ability to cope with the pandemic would increase in the next 12 months.

**Conclusions:**

This study confirmed a strong and negative impact on the mental health of the past 2 years of COVID-19 pandemic in the Italian population. Providing psychoeducation to the general population and improving the availability of telemedicine services could reduce the impact of future challenges related to the pandemic.

## Background

The COVID-19 pandemic resulted in an unprecedented global crisis that profoundly affected mental health and mental health care [1]. Prolonged isolation, uncertainty, and the temporary disruption of social relationships and interactions [2], negatively impacted the mental health and created new barriers. Depression, anxiety, and stress were reported as the most common mental health issues during the first waves of COVID-19, due to the prolonged quarantine, the completely new and unprecedented situation, and the higher exposure to information about the spread of COVID-19 and the associated mortality rates [3–7]. To reduce these negative effects, clinicians did their best to maintain support and treatment for their patients. For instance, telemedicine gradually became a vital resource for mental health care [8–10]. Also, new strategies and guidelines were recommended to help physicians manage patients during the acute phases of the pandemic, such as virtual psychological sessions able to reduce the psychological impact of COVID-19 in the general population and in certain categories of workers [11–15].

Italy was one of the first Western countries to be affected by the pandemic, and people spent more than 2 years facing unprecedented, new and often unpredictable challenges, often fluctuating between hope and optimism and worry and disappointment. Several studies reported the early psychological consequences of the pandemic COVID-19 in the Italian population [16, 17].

The impact of the pandemic on mental health has been less studied than the COVID-19 epidemiology, transmission patterns, physical symptoms, and their treatment [18]. Furthermore, while numerous studies have been conducted to assess physician and patient satisfaction with telemedicine [19–22], to our knowledge, fewer studies have been conducted to examine physicians’ perceptions of the mental health crisis and/or suggestions on how to best face the future challenges [1, 23–28].

The aim of this study was to survey a relatively large group of Italian physicians about their perceived impact of COVID-19 on the mental health of the Italian population and about their suggestions on the best way to address the future challenges.

To this end, we decided to conduct a survey among Italian clinicians to obtain an up-to-date overview of the current impact of the COVID-19 pandemic on the mental health of the Italian population and to gather their input on possible strategies to address the current and future challenges.

## Methods

A total of 1,281 clinicians practicing in Italy as psychiatrists, neuropsychiatrists, pediatricians, primary care practitioners, geriatricians, and neurologists participated in the survey, which was conducted between November 2021 and February 2022. The questionnaire was administered online through Google Form. Participation was voluntary, anonymous, and without remuneration. Written consent was obtained from all respondents. Given the nature of this survey, no ethics committee approval was required under Italian law.

The survey was developed by the authors of this paper in a series of consensus meetings to define the questions and make them clear and concise. In the case of disagreement, additional meetings were scheduled until full agreement was reached among all authors. The validity of the questionnaire was then tested on a sample of ten randomly selected psychiatric residents to assess the length of the survey and the appropriateness of the questions, and to ensure that the definitions were not ambiguous. The final version comprised four sections: (1) demographic data, including age, sex, and city of residence; (2) impact of the COVID-19 pandemic on mental health in their communities; (3) evaluation of the strategies that they followed to address the mental health impact of their patients; (4) current and planned strategies to respond and adapt to existing and future challenges.

Descriptive statistics were reported as mean ± standard deviation (SD) for quantitative data and as frequencies and percentages for qualitative data. A univariate Chi-square test was performed for comparisons among different specializations. Statistical significance was set at 5% (*p* < 0.05).

## Results

One thousand two hundred and eighty-one (1,281) clinicians with a mean experience of delivering health care of 23.03 ± 10.95 years participated in the survey (females: 551, males: 530, mean age 54.04 ± 11.01 years) (Table 1). The sample was mainly composed of adult and child (30.4% and 31.8%, respectively) psychiatrists, neurologists, primary care physicians, neurologists, and other specialists.

**Table 1:** Characteristics of survey respondents

Num. respondents	1281
Gender	
Females	551 (43.0%)
Males	730 (57.0%)
Specialization	
Psychiatrists or child neuropsychiatrists	389 (30.4%)
General practitioners	407 (31.8%)
Neurologists and geriatrics	244 (19.0%)
Not reported	241 (18.8%)
Respondents’ age* (years)	54.04 (11.01) (median: 58)
Experience* (years)	23.70 (10.95) (median: 27)

^*^Values are reported as mean (SD) and median

Eighty-one percent (*n* = 1,040) of respondents thought that the number of people with mental illness referring to their service had increased during the COVID-19 pandemic. Thirty-four percent reported that the number of people with a mental disorder in their community increased by 26–50%; about 33% reported an increase by 1–25%, and 26.9% reported an increase by 51–75%. Women were reported as more frequently hit by Covid-related mental distress than men (Table 2). When asked about which patients, in terms of age group, had been hit the most by mental health problems related to the Covid pandemic, 18.8% answered children and adolescents, 39% young adults, 33.6% adults, and 7.3% elderly subjects.

**Table 2:** Mental disease after COVID-19 outbreak

Do you think that the number of people with mental diseases has increased in the general population after the COVID-19 outbreak?	1040 (81.2%)
How much was the growth rate?	
1–25%	346 (33.3%)
26–50%	354 (34.0%)
51–75%	280 (26.9%)
76–100%	54 (5.2%)
Missing values	6 (0.6%)
In your experience, which gender has been hit the most by mental health problems related to the covid pandemic?	
Females	748 (58.4%)
Males	429 (33.5%)
Missing values or other	104 (8.1%)
In your experience, which of the following age groups has been hit the most by mental health problems related to the covid pandemic?	
Children and adolescents (≤ 18 years)	241 (18.8%)
Young adults (18–30 years)	500 (39.0%)
Adults (31–65 years)	431 (33.6%)
Elderly (≥ 65 years)	93 (7.3%)
Missing values	16 (1.2%)

Among the mental health conditions whose onset was related to the pandemic, agitation and mood disorders (especially anxiety) were the most frequently reported (by 62.3% and 31.5% of respondents, respectively), with similar distributions among different specialists (adult and child psychiatrists: geriatricians and neurologists: general practitioners and pediatricians) (Table 3).

**Table 3:** Most common symptom of psychiatric distress observed in your patients

	Total (*n* = 1040)	Adult and child psychiatrists (*n* = 389)	Geriatricians and neurologists (*n* = 244)	General practitioners and pediatricians (*n* = 407)
Agitation	648 (62.3%)	243 (62.5%)	143 (58.6%)	262 (64.4%)
Mood disorders	328 (31.5%)	122 (31.4%)	89 (36.5%)	117 (28.7%)
Eating disorders	64 (6.2%)	24 (6.2%)	12 (4.9%)	28 (6.9%)

**Fig. 1:**
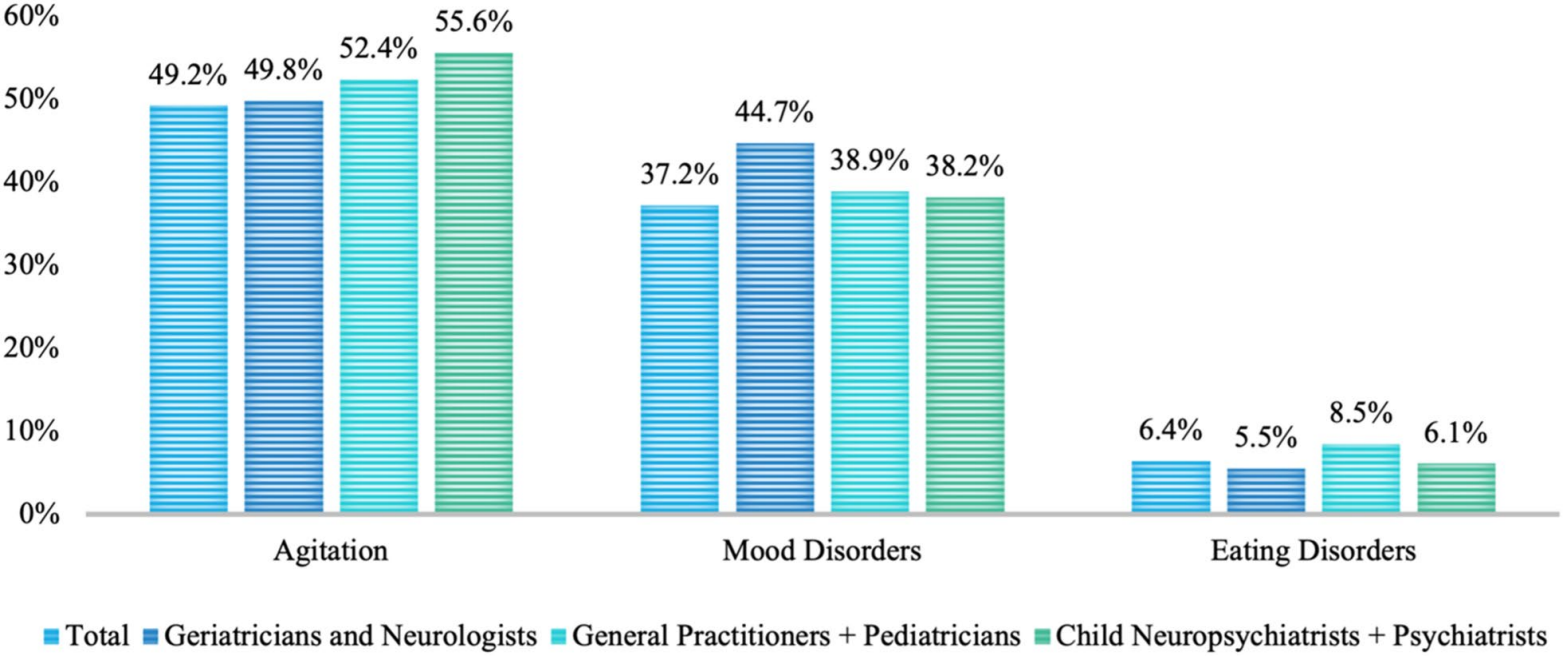
Pre-existing psychiatric condition that worsened the most

For 75.2% of respondents, the severity of pre-existing psychiatric conditions worsened during the pandemic. When asked about which condition/disease declined the most, agitation and mood disorders were the most frequently reported conditions (Fig. 1).

During the pandemic, 42.1% of respondents treated mental health diseases mainly with face-to-face visits, while 48.5% provided medical services via phone calls, video calls, or e-mail (Table 4).

**Table 4:** Strategies adopted during the pandemic—which strategies did you use the most during the pandemic to treat your patients with mental health problems?

Face-to-face visits	539 (42.1%)
Tele-medicine	621 (48.5%)
Extension of personal and/or telephone availability	121 (9.4%)

**Fig. 2:**
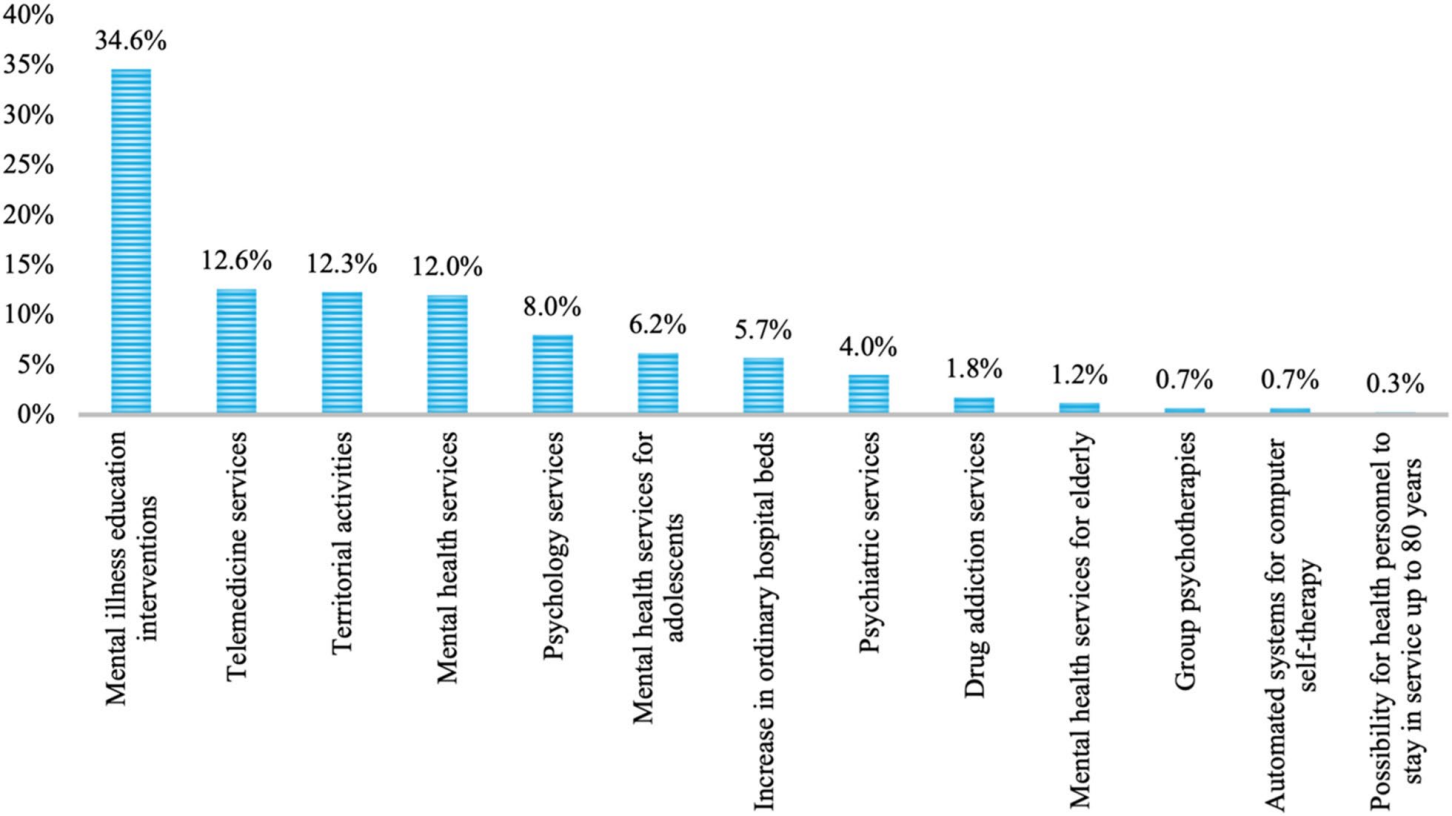
Most important strategies to address future challenges related to the COVID pandemic

Regarding the suggested strategies to address future challenges related to the COVD pandemic, 34.6% of respondents recommended providing psychoeducation to the general population for early recognition of mental diseases and teaching strategies for reducing the impact of Covid-related distress. In addition, 12.6% suggested an improvement in telemedicine services, while 12.3% suggested an increase in the resources allocated to community-based care (Fig. 2).

**Fig. 3:**
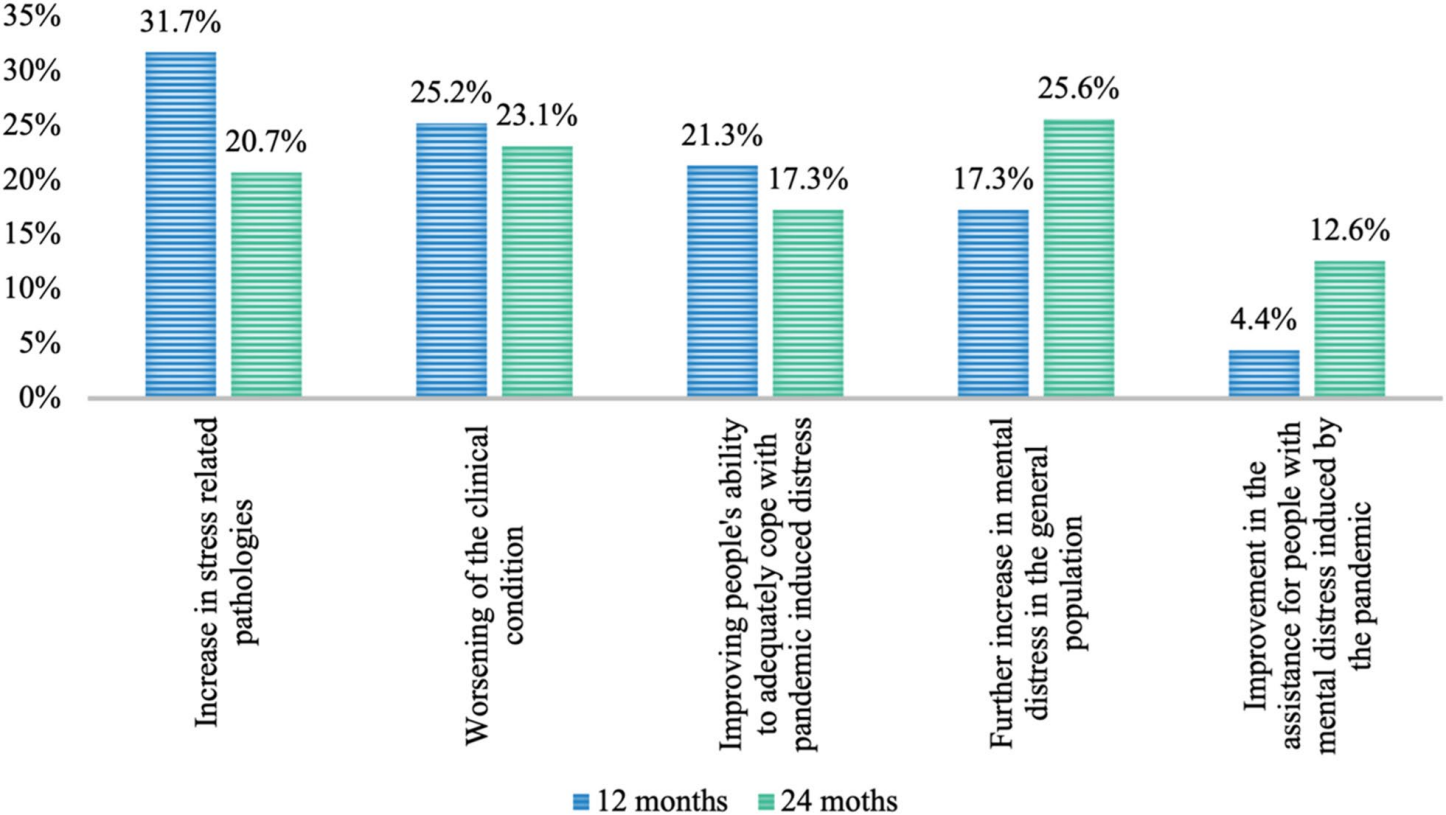
Most likely scenario in the next 12–24 months

When asked about their opinion regarding the trend of mental illness over the next 12 months, more than 30% predicted an increase in stress-related diseases, while 25.2% were more worried about worsening patients’ clinical conditions with previous psychiatric disorders. However, 21% of respondents believed that people’s ability to cope with the pandemic distress would increase over the next 12 months (Fig. 3).

## Discussion

In Italy, after more than 2 years from the first COVID-19 case (February 2020), a sense of uncertainty and fear has remained about the SARS-CoV-2 pandemic.

More than 80% of interviewees reported an increase in mental distress among their patients. Agitation and anxiety were reported among the most prevalent psychological burdens. From recent epidemiological studies, anxiety and depression related to the COVID-19 outbreak were reported worldwide in the general population with a prevalence of 33% (95%CI 28–38%) and 28% (95%CI 23–32%), respectively [29]. According to Talevi et al. (2020), who reviewed a series of 15 Chinese studies about mental health outcomes of the COVID-19 pandemic, in China, 7–58% of the non-clinical population experienced psychological distress at the initial stage of the COVID-19 outbreak with a range of negative responses, like anxiety, depression, and stress, together with other conditions as insomnia, worries about own health or family, phobias, and physical symptoms [26]. Analyzing the causes of such issues, people’s media overexposure and financial and social uncertainty appeared to be the most likely reasons for increased agitation, as emerged in previous major disasters [30]. From SARS in 2003 to H1N1 in 2010, all infectious outbreaks accompanied heavy psychological burdens, such as agitation, anxiety, depression, or panic attacks [31, 32]. Still, the role of the social context increased the sense of uncertainty considerably. The negative effects may have been further boosted by some factors shared by the entire population, such as the global spread of the pandemic, prolonged quarantines, lack of an effective treatment against COVID-19 (only recently in Italy are present specific pharmacological treatments for SARS-CoV-2), and uncertainty due to the lack of knowledge of vaccination coverage, and disinformation and false reports. For example, on this last topic, since the first vaccine campaign was promoted at the beginning of 2021, the Italian population has been dealt with a series of fake news about COVID-19 vaccines and their possible adverse effects: erroneous information about a high risk of mortality after vaccine injection or use of dangerous substances in vaccine formulations have prevented many Italians from vaccination, generating more increased agitation and anxiety levels. In this context, social media played a crucial role in increasing psychological distress: from our findings, the most exposed mediatic population, like women and young adults, were more subjected to psychological burdens. According to the results of Gao et al. [33], a high prevalence of mental health problems is positively associated with frequent social media exposure, called infodemic: about 82% of participants frequently exposed to social media showed high odds of anxiety and a combination of anxiety and depression [33].

Contrary to other studies, Italian clinicians did not report a high prevalence of psychological distress among the elderly: this result appears encouraging for the Italian healthcare system but needs to be interpreted very cautiously, in light of the limitations described below. From the beginning of the COVID-19 outbreak, the Italian government and, by consequence, the whole healthcare system have adopted a series of precautions to protect the weakest population: older people were advised to remain at home and protect themselves by avoiding interaction with non-cohabiting family members. These precautions may have increased the sense of protection of the elderly, who better respond in terms of psychological distress to the subsequent COVID-19 waves.

According to our findings, patients with previous mental disorders were particularly affected by the COVID-19-related psychological distress. Increased levels of anxiety and agitation were noted among the general population, but were even more severe in patients with a previous history of psychological or psychiatric distress/disease.

Many interviewees preferred to maintain face-to-face visits at the first onset of mental distress and during the follow-up period. However, many other resources such as video calls or remote psychotherapy were adopted. As for the trends and predictions for the future of mental health, an increase in all stress-related pathologies and a worsening of clinical conditions are expected from many clinicians within 12–24 months. Despite these negative expectations, clinicians believe that an improvement in people’s ability to cope with pandemic-induced stress will also be observed.

This study has some limitations. First, it was conducted on clinicians and not on their patients, so epidemiological conclusions for the actual impact of COVID-19 after the acute pandemic phase were not possible. Second, responses were collected during the fourth COVID-19 wave characterized by the prevalence of a new variant (Omicron), about which a higher diffusion capacity than the Delta variant and an initial low level of knowledge about omicron negative effects could have incremented the request for psychological support. Combined, these factors could have intensified interviewees’ perception of patients’ psychological discomfort. Third, this study, reflecting physicians’ views, could also be defined as a qualitative study, with all the limitations that arise from this type of studies. Indeed, our sample was not necessarily representative of the thousands of doctors who practice in Italy. Furthermore, given that participants were recruited through E-mails and social media, we cannot exclude a sampling bias that would compromise the validity of the study. Finally, physician burnout was not controlled for and this further limits the validity of our survey, given that clinicians’ opinions on patients' mental health is likely also influenced by the mental health and general level of physician burnout.

## Conclusions

Our study has shown the remarkable capability of Italian clinicians to adopt new strategies to manage an escalating request for psychological and psychiatric support. The COVID-19 pandemic has presented an unprecedented scenario for the mental health in the general population and for mental health providers. These lasts appear well cognizant of the past, current and future challenges, but also relatively confident on the ability to face them at the best, especially if strategies and tools such as psychoeducation and telemedicine are correctly implemented and funded.

## Data Availability

The datasets used and/or analyzed during the current study are available from the corresponding author upon request.
